# Benchmarking Lightweight YOLO Object Detectors for Real-Time Hygiene Compliance Monitoring

**DOI:** 10.3390/s25196140

**Published:** 2025-10-04

**Authors:** Leen Alashrafi, Raghad Badawood, Hana Almagrabi, Mayda Alrige, Fatemah Alharbi, Omaima Almatrafi

**Affiliations:** 1 Department of Information Systems, Faculty of Computing and Information Technology, King Abdulaziz University, Jeddah 21589, Saudi Arabia; 2 Department of Computer Science, College of Computer Science and Engineering, Taibah University, Yanbu 46421, Saudi Arabia

**Keywords:** computer vision, deep learning, hygiene compliance, object detection, personal protective equipment (PPE), real-time monitoring, benchmarking, model efficiency, IoT-integrated systems

## Abstract

Ensuring hygiene compliance in regulated environments—such as food processing facilities, hospitals, and public indoor spaces—requires reliable detection of personal protective equipment (PPE) usage, including gloves, face masks, and hairnets. Manual inspection is labor-intensive and unsuitable for continuous, real-time enforcement. This study benchmarks three lightweight object detection models—YOLOv8n, YOLOv10n, and YOLOv12n—for automated PPE compliance monitoring using a large curated dataset of over 31,000 annotated images. The dataset spans seven classes representing both compliant and non-compliant conditions: glove, no_glove, mask, no_mask, incorrect_mask, hairnet, and no_hairnet. All evaluations were conducted using both detection accuracy metrics (mAP@50, mAP@50–95, precision, recall) and deployment-relevant efficiency metrics (inference speed, model size, GFLOPs). Among the three models, YOLOv10n achieved the highest mAP@50 (85.7%) while maintaining competitive efficiency, indicating strong suitability for resource-constrained IoT-integrated deployments. YOLOv8n provided the highest localization accuracy at stricter thresholds (mAP@50–95), while YOLOv12n favored ultra-lightweight operation at the cost of reduced accuracy. The results provide practical guidance for selecting nano-scale detection models in real-time hygiene compliance systems and contribute a reproducible, deployment-aware evaluation framework for computer vision in hygiene-critical settings.

## 1. Introduction

Maintaining hygiene in shared workspaces—such as food preparation areas, healthcare facilities, and industrial environments—is essential for public health, operational safety, and regulatory compliance. According to the World Health Organization, foodborne illnesses alone affect approximately 600 million people each year, resulting in over 420,000 deaths globally [1]. A significant proportion of these cases are due to hygiene practices, including the inconsistent or improper use of personal protective equipment (PPE) such as gloves, face masks, and hairnets.

Ensuring adherence to PPE protocols remains a persistent challenge, particularly in high-throughput, fast-paced, or semi-structured environments. Manual inspections, though still widely used, are inherently labor-intensive, error-prone, and unsuitable for continuous real-time enforcement. These limitations highlight the need for intelligent, automated systems capable of detecting hygiene compliance violations accurately and efficiently.

Recent advances at the intersection of computer vision and the Internet of Things (IoT) have created new opportunities for scalable hygiene monitoring solutions. In smart environments equipped with cameras and edge computing devices, deep learning-based object detection models can be deployed to monitor PPE use in real time, thereby reducing reliance on manual inspection and supporting more consistent enforcement of hygiene standards. Among these models, one-stage detectors—particularly those in the You Only Look Once (YOLO) family—are known for their balance of accuracy, speed, and model compactness, making them well-suited for deployment on edge devices.

While YOLO-based architectures have been applied in fields such as agriculture [2], transportation [3], and general kitchen safety [4], limited research has explored their effectiveness for PPE compliance detection in hygiene-critical environments, where multiple objects need to be detected simultaneously. In particular, few studies have systematically benchmarked modern object detection architectures for both correct and incorrect PPE use, especially with attention to practical deployment in regulated settings. This remains an important and understudied area in computer vision research [5,6].

To address this gap, this study evaluates and compares the performance of three deep learning-based lightweight object detectors—YOLOv8n, YOLOv10n, and YOLOv12n—on a curated dataset of more than 31,000 annotated images. The study addresses the following three research questions:

1. Which object detection model offers the best trade-off between detection accuracy and deployment efficiency for real-time PPE compliance monitoring in IoT-integrated environments?
2. How does detection performance vary across PPE categories (e.g., gloves, hairnets, masks, incorrect mask), and are some classes consistently more challenging to detect?

All evaluations are conducted using standardized deployment-relevant metrics, including mean Average Precision (mAP@50 and mAP@50–95), inference speed (Frames Per Second, FPS), and model complexity (parameter count and GFLOPs). These analyses are designed to inform the development of real-time, IoT-integrated hygiene monitoring systems for safety-critical environments.

Key Contributions
  - We curate an annotated domain-specific dataset of over 31,000 images for hygiene compliance detection collected from commercial kitchens, food processing facilities, and hospitals, with seven PPE-related categories: *glove*, *no glove*, *hairnet*, *no hairnet*, *mask*, *no mask*, and *incorrect mask*. The dataset is publicly available to enable reproducibility and fair benchmarking.
  - We benchmark three nano-scale YOLO models (YOLOv8n, YOLOv10n, YOLOv12n) in the domain-specific context of PPE compliance monitoring, evaluating both accuracy (mAP, precision, recall) and deployment-aware efficiency (FPS, parameter count, GFLOPs).
  - We report model accuracy–efficiency trade-offs and class-wise challenges that inform real-time, IoT-enabled compliance systems in hygiene-regulated environments.

## 2. Related Work

### 2.1. Real-Time Object Detection Models for IoT-Enabled Monitoring

Real-time object detection is a key element in developing IoT-enabled monitoring systems. Its main role is to allow devices to automatically perceive and interpret their surroundings, enabling quick and accurate responses with low latency. This ability is essential in many fields, including industrial safety monitoring [7,8], industrial automation [9], and aviation for real-time airplane detection [10], where the speed and accuracy of detection directly influence efficiency and safety.

Recent advances have focused on optimizing deep learning models—particularly those based on convolutional neural networks (CNNs) and one-stage detectors such as the YOLO (You Only Look Once) family—for deployment on resource-constrained IoT devices. One-stage detectors perform object localization and classification in a single network pass, making them especially suitable for real-time applications because of their high inference speed and relatively low computational demands compared to two-stage methods like Faster R-CNN [11,12]. As a result, these models balance detection accuracy, computational efficiency, and response speed, making them ideal for edge-based IoT monitoring. In this study, we focus on YOLOv8n, YOLOv10n and YOLOv12n, which are widely adopted for real-time applications due to their ability to process entire images in a single forward pass, enabling efficient deployment on edge devices and embedded systems.

YOLOv10n represents the ultra-lightweight variant of the YOLOv8 family, designed for deployment on edge and IoT devices where latency and memory efficiency are critical. It employs an anchor-free, decoupled detection head in combination with a CSP-based backbone and a PAN/FPN-style neck to enable effective multi-scale feature fusion [13]. These architectural choices enhance both efficiency and accuracy, while the model also provides streamlined support for deployment frameworks such as ONNX and TensorRT. In practice, YOLOv8n achieves millisecond-level inference speeds with competitive accuracy, for example, reporting approximately 80.9% mAP in strawberry instance segmentation tasks, thereby demonstrating a favorable balance between speed and accuracy under resource constraints [14].

YOLOv10n extends this efficiency-driven design philosophy by introducing a non-maximum suppression (NMS)-free training strategy, enabled through consistent dual label assignments that combine one-to-many and one-to-one matching. Together with an optimized CSP backbone and PAN neck, this approach improves both detection stability and runtime efficiency. On the COCO benchmark, YOLOv10n achieves 38.5 AP (50–95) with a latency of only 1.84 ms on an NVIDIA T4 GPU (Santa Clara, CA, USA) using TensorRT FP16, highlighting its strong accuracy–efficiency trade-off in the nano configuration [15].

YOLOv12n advances toward an attention-oriented architecture while retaining real-time performance. It incorporates Area Attention, R-ELAN (Residual Efficient Layer Aggregation Network), and FlashAttention, combined with lightweight design choices such as reduced MLP ratios and a 7 × 7 separable position perceiver for efficient positional encoding. Evaluated on COCO val2017, YOLOv12n reports 40.6 AP (50–95) with an inference latency of 1.64 ms under TensorRT FP16 on an NVIDIA T4 GPU. This performance indicates modest accuracy gains compared to earlier nano versions while maintaining comparable inference speed, making it a strong candidate for real-time, resource-constrained deployment scenarios [16].

As object detection models are increasingly deployed on edge and IoT systems in industries such as construction and manufacturing, deployment-oriented factors—such as model size, inference latency, and computational efficiency—have become critical [9]. However, as highlighted in a recent systematic review, few studies in the context of PPE compliance systematically evaluate both detection accuracy and operational efficiency, limiting their practical applicability in real-world settings [5].

### 2.2. PPE Compliance Detection in Hygiene and Safety-Critical Settings

Computer vision-based PPE compliance monitoring has gained attention in safety-critical domains. A recent review by Vukicevic et al. highlights persistent technical challenges, including PPE design variability, visual clutter, occlusion, and lighting variation [5]. YOLO-based detectors have significantly improved detection accuracy in many contexts, but their performance often declines in semi-structured or dynamic environments such as kitchens or hospitals.

Most progress in PPE detection has occurred in the construction sector, where visual conditions are relatively structured. For example, Ferdous and Ahsan trained a YOLO-based model on the CHVG dataset and achieved 89.84% mAP across eight PPE classes [17]. Biswas and Hoque reported 95.4% mAP for mask detection using a YOLOv8-based system, with inference speeds exceeding 150 FPS [7]. Other studies, such as [6,8], have used YOLOv8 in UAV-based or outdoor detection systems to identify safety gear under variable lighting and weather conditions, demonstrating strong generalization and scalability.

In contrast, hygiene-critical domains such as food preparation and healthcare have received far less attention. Zhou et al. implemented a YOLOv5s model on an NVIDIA Jetson Xavier NX to detect hats and masks in kitchen environments, achieving 85.7% average precision at 31 FPS [18]. However, the system’s performance declined when detecting transparent or non-standard PPE. In addition, Guo et al. used YOLOv5l with DeepSort for staff behavior monitoring in kitchens, but their focus was on general activity tracking rather than detailed compliance evaluation [19].

More recently, a study compared YOLOv5, YOLOv8, and YOLOv10 for kitchen safety applications, reporting that YOLOv8 performed best in fine-grained tasks such as gesture recognition [4]. Another study evaluated YOLOv6, YOLOv8, and DenseNet for recognizing handwashing actions and PPE usage among food handlers [20]. However, both studies were limited in scope, focusing on specific actions within the food preparation process and relying primarily on standard accuracy-based evaluation metrics.

Despite recent advances, as emphasized by Vukicevic et al. [5], there remains a significant lack of publicly available, domain-specific datasets targeting hygiene-related PPE such as hairnets, disposable gloves, and masks. Most existing datasets and models focus on industrial PPE like hard hats and safety vests, which have distinct visual characteristics, while the nuanced appearance of hygiene gear—often small, deformable, or transparent—is poorly represented in current benchmarks. This limits both the training of robust models and the evaluation of generalization in real-world food safety environments.

These limitations underscore the need for systematic benchmarking of modern object detectors in hygiene-critical scenarios using representative datasets and deployment-oriented metrics. To address this gap, the present study evaluates three representative object detection models—YOLOv8n, YOLOv10n, and YOLOv12n—on a large, multi-context dataset. All models are assessed using both accuracy metrics (mAP@50, mAP@50–95) and deployment-relevant efficiency metrics (FPS, parameter count, and GFLOPs), providing practical guidance for scalable hygiene monitoring in IoT-enabled, safety-critical environments.

## 3. Materials and Methods

**Figure 1:**
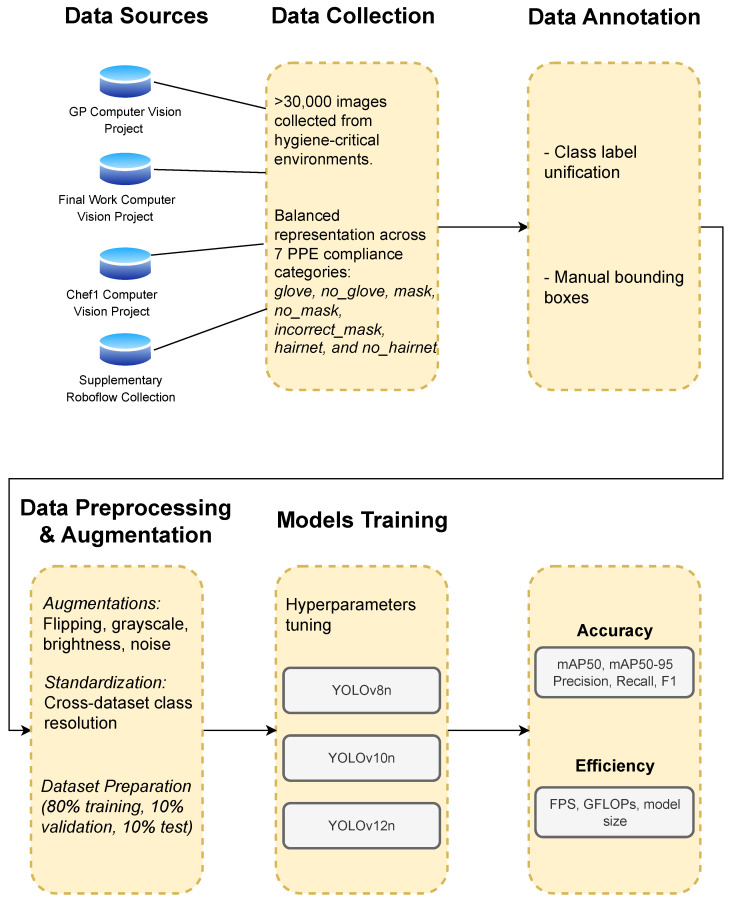
Methodological pipeline showing data collection from Roboflow sources [21,22,23], annotation, preprocessing, model training, and evaluation steps for real-time hygiene compliance detection in IoT-integrated environments.

The methodology (illustrated in Figure 1) comprises five key stages: data collection, annotation and label harmonization, preprocessing and augmentation, model training, and performance evaluation.

Model performance is assessed descriptively using standard object detection metrics—including mean Average Precision (mAP@50 and mAP@50–95), precision, recall, F1 score, inference speed (FPS), parameter count, and GFLOPs. These metrics are widely adopted in the computer vision literature and provide a comprehensive view of each model’s accuracy and deployment feasibility. No inferential statistical testing is applied, as the goal is not to generalize beyond the fixed dataset but to compare models under controlled, reproducible conditions aligned with real-world deployment scenarios.

### 3.1. Dataset Collection and Sources

**Figure 2:**
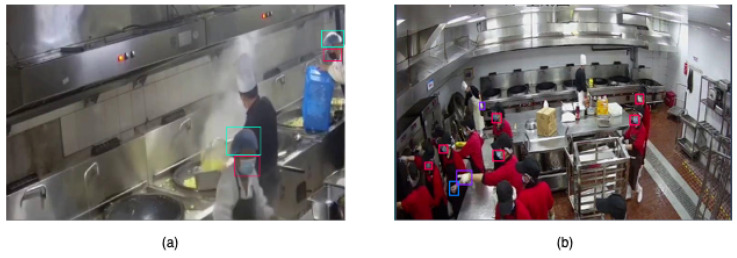
Example annotations from kitchen environments illustrating diverse visual conditions: (**a**) steamy workspace with occlusions and low visibility; (**b**) crowded kitchen captured with a wide-angle camera, highlighting multiple overlapping personnel and varied PPE compliance states.

To support various deployment scenarios in hygiene compliance monitoring, we curated the dataset by aggregating images from multiple publicly available sources, representing real-world visual contexts including commercial kitchens, food processing facilities, and hospitals. These environments were selected to capture a wide range of visual variability—including differences in lighting, occlusion, camera perspectives, and PPE types—that can significantly affect detection accuracy in hygiene-focused, IoT-enabled monitoring systems. Overall, while the images were sourced from publicly available Roboflow projects, every subset in the final dataset underwent manual review and correction to ensure high-quality annotations and class consistency (see Figure 2).

The following datasets, sourced from Roboflow projects, form the basis of our final dataset:

- *GP Computer Vision Project* [21]: Contains 7515 images with six labels: glove, hairnet, maskoff, maskon, no_glove, and no_hairnet. Images were captured from indoor environments, including kitchens and offices. Minor preprocessing was applied, including auto-orientation and ±7% brightness variation at the bounding box level.
- *Final Work Computer Vision Project* [22]: Includes 8283 images across six labels: gloves, hairnet, mask, no_mask, no_gloves, and no_hairnet. Captured in diverse environments (e.g., streets, kitchens). Preprocessing included auto-orientation and resizing to 640 × 640 pixels. Augmentations include horizontal/vertical flipping, 90° rotations, grayscale (14% of images), saturation adjustments (±22%), exposure shifts (±10%), and noise addition (up to 1.25% of pixels).
- *Chef1 Computer Vision Project* [23]: Consists of 5387 images captured exclusively in commercial kitchens. Originally labeled with four classes: clothes, correct_mask, hat, and human. For consistency, hat was relabeled as hairnet, and irrelevant classes (human, clothes) were discarded.
- *Supplementary Roboflow Collection*: An additional 7923 images were curated from Roboflow based on the presence of incorrect mask usage (e.g., worn below the nose/chin), with sources ranging from hospitals to schools and public areas. The inclusion of an ‘incorrect_mask’ category is motivated by the observation that most PPE detection work focuses solely on presence—e.g., “Is the item present?”—rather than correct usage. Vukicevic et al. [5] note that “only a few studies have addressed the issue of improper use of PPE,” which underscores the need to model misuse cases.

The dataset was reviewed to ensure that duplicate and null (no human or empty) images were removed.

### 3.2. Data Annotation and Label Harmonization

Although initial annotations existed for most datasets, we conducted a uniform manual review across the entire dataset, to address class imbalance, particularly for “incorrect_mask.” Annotation and correction were performed using the Roboflow platform, ensuring accurate identification of hygiene violations and clear distinction between absent and improperly worn PPE.

To ensure label consistency, we performed synonym resolution and remapping across datasets:

- “maskon” and “mask” were unified as mask.
- “maskoff” was resolved to no_mask or incorrect_mask, depending on visual context.
- Non-PPE or irrelevant labels (e.g., “person”, “clothes”) were removed.

These steps ensured uniform label semantics, critical for reliable model training and evaluation.

### 3.3. Data Preprocessing and Augmentation

All images were resized to 640 × 640 pixels, the standard input size for YOLO-based models. This resolution provides a practical balance between detection accuracy and computational efficiency, making it suitable for real-time inference on edge devices or smart camera platforms.

Existing augmentations in the original datasets were retained, including grayscale conversion, flipping, brightness/saturation adjustments, and noise. These transformations improve feature diversity and resemble real-world scenarios.

The final merged dataset includes 31,371 images across seven hygiene-related classes: glove, no glove, hairnet, no hairnet, mask, incorrect mask, and no mask. Table 1 summarizes the per-class distribution based on both image and instance counts. All dataset curation, annotation harmonization, and class mapping were completed prior to any model training or evaluation. The final dataset split (80/10/10) was fixed before initiating experiments, and no information from the test set was used during model development or parameter tuning.

### 3.4. Model Selection and Training Protocol

In this study, YOLOv8n, YOLOv10n, and YOLOv12n were selected as the candidate models for comparative evaluation. The focus on the nano variants is motivated by the deployment-aware nature of hygiene monitoring applications, where inference speed, computational efficiency, and memory footprint are critical constraints. These models represent successive generations of the YOLO family, each introducing architectural refinements and efficiency improvements while maintaining competitive detection accuracy. YOLOv8-nano serves as a strong baseline widely adopted in practice, YOLOv10-nano incorporates design modifications aimed at optimizing the accuracy–efficiency balance, and YOLOv12-nano reflects the most recent advancements in lightweight object detection. Together, these selections provide a representative and up-to-date spectrum of real-time detectors suitable for resource-constrained deployment scenarios.

All models were trained on the same dataset split using consistent training procedures to ensure fair comparison. Key training parameters included the following:

- Epochs: 60.
- Batch Size: 32.
- Learning Rate: 0.01.
- Input Size: 640 × 640 pixels.
- Initialization: Pretrained weights.
- Frozen Layer: model.23.dfl.conv.weight was frozen to stabilize distribution focal loss computation, enhancing convergence on class-imbalanced data.

#### Computing Infrastructure

All experiments, including those involving the YOLOv8n, YOLOv10n, and YOLOv12n models, were conducted in Google Colab using an NVIDIA Tesla T4 GPU (16 GB VRAM), 52 GB RAM, and an Intel Xeon CPU (Santa Clara, CA, USA) backend running Ubuntu 22.04.4 LTS. The implementation environment used Python 3.11 with relevant libraries: Ultralytics YOLOv8.2.103 for YOLOv8n; the official Tsinghua YOLOv10 repository for YOLOv10n; and Ultralytics 8.3.63 Python-3.12.11 torch-2.8.0 torchvision-0.20.0 for YOLOv12n. All models were trained and evaluated with CUDA 12.4 or 12.5 and cuDNN enabled for GPU acceleration. To ensure a fair comparison, training and inference were performed under consistent conditions.

### 3.5. Evaluation Metrics and Deployment Considerations

This study evaluates model performance in two dimensions: detection accuracy and deployment feasibility. This dual emphasis reflects best practices in object detection benchmarking, particularly for real-time, resource-constrained applications such as IoT-integrated hygiene monitoring systems [24,25].

#### 3.5.1. Detection Accuracy Metrics

To assess the correctness and robustness of predictions, we use several standard evaluation metrics widely adopted in the computer vision literature:

- **Mean Average Precision (mAP)**
  The primary metric for evaluating object detection performance. mAP is computed as the mean of Average Precision (AP) across all object classes. AP reflects the area under the precision–recall curve, capturing both false positives and false negatives.
  – mAP@0.50 (mAP50): Measures detection performance at a fixed Intersection over Union (IoU) threshold of 0.50. It is commonly used for baseline comparisons and coarse localization assessment [24].
  – mAP@0.50:0.95 (mAP50–95): A stricter metric averaging AP across IoU thresholds from 0.50 to 0.95 in 0.05 increments. It provides a more comprehensive view of model performance under varying localization difficulty [24].
- **Precision and Recall**
  Precision reflects the proportion of correct positive detections, while recall captures the proportion of true objects successfully detected. These metrics are critical in safety-sensitive applications, where both false positives (e.g., falsely detecting PPE when absent) and false negatives (e.g., missing a violation) may have operational consequences.

#### 3.5.2. Deployment Efficiency Metrics

In addition to detection accuracy, we report metrics that characterize each model’s suitability for real-time deployment:

- **Inference Speed (Frames Per Second, FPS):** FPS measures how many frames a model can process per second. It directly impacts responsiveness and system throughput. High FPS values are necessary for real-time applications in high-traffic environments such as commercial kitchens or healthcare facilities.
- **Computational Complexity (GFLOPs):** GFLOPs quantify the number of floating-point operations required per image. Lower GFLOPs typically indicate better suitability for embedded or resource-constrained platforms, although they may involve trade-offs with detection precision.
- **Model Size (Parameters):** We also report parameter count as an indicator of model size and memory footprint. This metric is especially relevant for deployment on devices with limited storage or transfer bandwidth.

These metrics collectively reflect the trade-offs between detection accuracy, computational cost, and real-time inference feasibility. This approach aligns with benchmarking practices seen in deployment-aware studies such as [25], which emphasize joint reporting of accuracy (e.g., AP) and efficiency (e.g., GFLOPs, FPS) to guide real-world integration.

Although this study does not include physical hardware deployment, all evaluation procedures and metrics were selected with embedded systems in mind, enabling reproducible and practical guidance for smart PPE compliance monitoring in IoT-enabled environments.

## 4. Results

This section presents the benchmarking results and key observations from the evaluation of three nano-scale object detection models—YOLOv8n, YOLOv10n, and YOLOv12n—on a multi-class hygiene compliance dataset. The evaluation focuses on detection accuracy, inference efficiency, model learning behavior, and deployment feasibility in IoT-integrated environments, with particular attention to balancing accuracy and computational efficiency for real-time applications.

We first compare global detection metrics across the three models, followed by a per-class performance analysis to highlight category-specific strengths and weaknesses. Finally, we examine training and validation loss trends to assess learning behavior and generalization capacity.

### 4.1. Model Performance Benchmarking

Table 2 presents a comparative evaluation of YOLOv8n, YOLOv10n, and YOLOv12n across detection accuracy, inference speed, and model complexity metrics on the test set. In terms of precision, YOLOv8n achieved the highest value (84.3%), followed closely by YOLOv10n (83.9%), while YOLOv12n showed a modest decline (80.5%). For recall, YOLOv8n again led with 80.7%, compared to 79.8% for YOLOv10n and 74.7% for YOLOv12n. A similar trend was observed in the detection accuracy metrics: YOLOv10n obtained the best mAP@50 (85.7%), slightly surpassing YOLOv8n (85.6%), whereas YOLOv12n lagged at 80.8%. For the more challenging mAP@50–95 metric, YOLOv8n performed best (55.7%), followed by YOLOv10n (54.8%), while YOLOv12n trailed with 49.2%.

In terms of inference speed, YOLOv10n achieved the highest throughput at 381.15 FPS, followed by YOLOv8n at 336.30 FPS, while YOLOv12n was the slowest at 277.89 FPS despite being the smallest model. This highlights that model size alone does not guarantee faster runtime efficiency, as architectural differences and computational design also play key roles.

From a deployment perspective, YOLOv12n offers the smallest model size (2.5 M parameters) and lowest computational cost (5.8 GFLOPs), making it lightweight and potentially suitable for resource-constrained devices. However, this efficiency comes at the expense of accuracy, as YOLOv12n consistently underperformed compared to the other two models. YOLOv10n, while slightly larger (2.7 M parameters, 8.2 GFLOPs), demonstrated the best balance between detection accuracy and efficiency, achieving competitive precision and the highest mAP@50. YOLOv8n, with a size of 3.0M parameters and 8.1 GFLOPs, attained the best recall and mAP@50–95, making it the strongest performer in terms of robust detection across stricter IoU thresholds, though with marginally higher computational demand.

Overall, YOLOv10n appears the most deployment-ready for real-time hygiene compliance monitoring in IoT-integrated environments, offering an effective trade-off between accuracy and computational efficiency, while YOLOv8n provides higher robustness for stricter detection requirements, and YOLOv12n favors lightweight scenarios where resources are severely limited.

### 4.2. Class-Wise Detection Performance

Table 3 summarizes the per-class detection performance of YOLOv10n across eight PPE-related categories. The model achieved strong overall accuracy, with an aggregate precision of 83.9%, recall of 79.8%, and mAP@50 of 0.857. Among the individual classes, hairnets were the most reliably detected item, both in terms of presence and absence. The hairnet class achieved the highest recall (93.8%) and the best overall localization accuracy (mAP@50–95 = 0.683), while no_hairnet also performed strongly (mAP@50–95 = 0.599). This consistency suggests that hairnets are particularly well-suited for detection due to their clear visual cues, minimal occlusion, and strong contrast between covered and uncovered hair. Gloves were also detected with relatively high accuracy (mAP@50–95 = 0.628), confirming YOLOv10n’s robustness in identifying well-defined PPE items.

In contrast, mask-related categories remained more challenging. The mask class recorded a comparatively low localization accuracy (mAP@50–95 = 0.473), indicating reduced precision in bounding box placement. Incorrect_mask achieved slightly better localization (0.545) but still lagged behind PPE categories with stronger visual distinctiveness. The no_mask class had the lowest overall localization accuracy (mAP@50–95 = 0.448), underscoring the difficulty of reliably detecting mask absence when cues are subtle, variable, or obstructed.

Detection of non-compliance categories (no_hairnet, no_glove, no_mask) was generally weaker than that of PPE-present classes. Among these, no_hairnet achieved the strongest results, with recall of 85.5% and mAP@50–95 of 0.599, likely due to the distinct contrast between covered and uncovered hair. In comparison, no_glove (mAP@50–95 = 0.458) and no_mask (0.448) were more difficult to detect, possibly due to hand and face occlusions or higher variability in appearance. These findings indicate that identifying PPE absence or misuse remains more challenging than detecting its correct presence.

Overall, while YOLOv10n demonstrated competitive performance across most categories, mask-related and non-compliance classes remain the most difficult to detect. These results suggest that targeted training strategies, class-specific data augmentation, or specialized detection modules could further enhance performance in hygiene-critical monitoring applications.

### 4.3. Training and Validation Loss Trends

**Figure 3:**
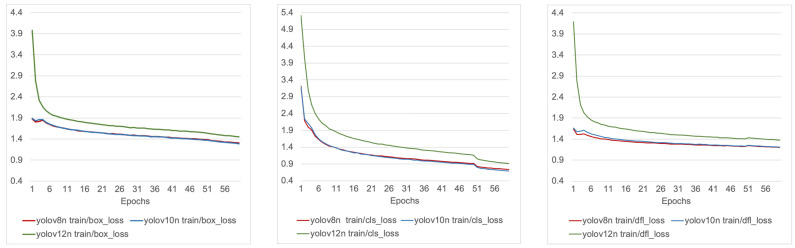
Training loss comparison for YOLOv8n, YOLOv10n, and YOLOv12n.

**Figure 4:**
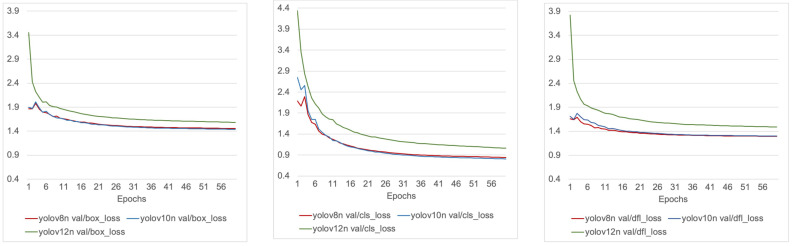
Validation loss comparison for YOLOv8n, YOLOv10n, and YOLOv12n.

Figure 3 and Figure 4 illustrate the training and validation loss trajectories for YOLOv8n, YOLOv10n, and YOLOv12n across the three primary loss components: bounding box regression loss (box_loss), which measures the discrepancy between predicted and ground-truth bounding box coordinates; classification loss (cls_loss), which penalizes incorrect category predictions; and distribution focal loss (dfl_loss), which enhances localization accuracy by modeling bounding box offsets as discrete probability distributions rather than direct scalar values.

During training (Figure 3), YOLOv8n and YOLOv10n consistently converged faster and stabilized at lower final loss values compared to YOLOv12n. Their box and classification losses followed nearly overlapping trajectories, while YOLOv8n maintained a slight advantage in distribution focal loss, suggesting stronger localization performance. YOLOv12n, by contrast, retained higher loss values across all components throughout training, indicating less efficient optimization.

The validation curves (Figure 4) mirrored these trends. YOLOv8n achieved the lowest validation classification and localization losses, confirming its robustness under generalization conditions. YOLOv10n also converged well, reaching comparable levels for box and classification losses with only a modest gap in dfl_loss. YOLOv12n exhibited slower convergence and plateaued at higher validation losses across all components, pointing to weaker generalization capacity and underfitting relative to the other models.

Overall, the combined training and validation loss analysis indicates that YOLOv8n and YOLOv10n learn more effectively and generalize better than YOLOv12n. Between the two, YOLOv8n demonstrates stronger localization accuracy, while YOLOv10n provides a competitive balance of convergence stability and efficiency, aligning with their superior test set performance compared to YOLOv12n.

## 5. Discussion

### 5.1. Balancing Accuracy and Efficiency in Real-Time Systems

The benchmarking results confirm that model accuracy alone is insufficient when selecting object detectors for IoT-enabled systems. Among the nano-scale variants, YOLOv10n demonstrated the most balanced profile, achieving the highest mAP@50 (85.7%) with competitive precision and recall, while maintaining a lightweight architecture (2.7 M parameters, 8.2 GFLOPs). This combination of accuracy and efficiency makes YOLOv10n particularly suitable for deployment on embedded and edge devices, where constraints on memory, latency, and energy consumption are critical.

YOLOv8n, while slightly less efficient (3.0M parameters, 8.1 GFLOPs), achieved the strongest localization performance at stricter IoU thresholds (mAP@50–95: 55.7%) and the highest recall (80.7%). This suggests YOLOv8n may be preferable in scenarios where robust detection under challenging conditions or higher tolerance for subtle PPE violations is prioritized, even at the cost of marginally greater computational demand.

In contrast, YOLOv12n favored lightweight operation (2.5M parameters, 5.8 GFLOPs) but underperformed across accuracy metrics, converging more slowly during training and plateauing at higher validation losses. Its reduced detection reliability, particularly for mask-related and non-compliance categories, limits its suitability for high-stakes hygiene monitoring tasks, though it may still be considered in extremely resource-constrained environments where model size is the dominant factor.

### 5.2. Deployment Implications for Compliance Monitoring Systems

The comparative evaluation highlights YOLOv10n as the most deployment-ready candidate for real-time, on-device inference in PPE compliance systems. Its fast inference speed (381.15 FPS), compact size (2.7M parameters), and strong accuracy make it well-suited for integration into smart cameras, mobile IoT platforms, or edge gateways—enabling low-latency alerts, continuous monitoring, and automated compliance feedback in kitchens, hospitals, and other regulated environments.

YOLOv8n, while slightly larger and slower (336.30 FPS), demonstrated superior localization accuracy under stricter IoU thresholds (mAP@50–95 = 55.7%) and higher recall (80.7%). This makes it particularly valuable in scenarios where minimizing missed detections is more critical than maximizing throughput, such as in high-risk areas where PPE violations carry significant safety implications.

YOLOv12n, despite its ultra-lightweight design (2.5M parameters, 5.8 GFLOPs), offered the weakest accuracy and slower inference speed (277.89 FPS) compared to the other nano variants. Its limited reliability suggests that while it may be considered for extremely resource-constrained environments, it is less suitable for high-stakes hygiene compliance applications.

The comparative results suggest that architectural design choices may influence the balance between accuracy and efficiency. YOLOv8n, with its anchor-free, decoupled detection head and enhanced feature representation system, demonstrates strengths in localization and balanced overall performance. YOLOv10n incorporates NMS-free training with consistent dual label assignments and backbone refinements, which may explain its improved inference efficiency while retaining accuracy comparable to YOLOv8n. This outcome is consistent with prior reports that emphasize YOLOv10’s design focus on optimizing the accuracy–efficiency trade-off. YOLOv12n, while integrating attention-centric modules such as Area Attention, R-ELAN, and FlashAttention, emphasizes compactness and computational efficiency. However, in our experiments this design corresponded to reduced generalization compared with YOLOv8n and YOLOv10n. Taken together, these findings indicate that while architectural minimization and efficiency-oriented modules enhance deployment readiness, excessive compression may limit representational capacity, which is a critical consideration for safety-sensitive, deployment-aware applications.

This study underscores the importance of deployment-aware benchmarking for practical computer vision solutions. By jointly evaluating mAP, inference speed, parameter count, and computational load (GFLOPs), we provide a multidimensional framework for model selection that accounts for the operational constraints and safety requirements of hygiene-critical environments.

### 5.3. Generalizability and Future Work

Although this work focused on PPE items commonly used in food preparation environments, the proposed benchmarking protocol and insights generalize to other hygiene-regulated domains, such as pharmaceutical manufacturing and clinical laboratories. The dataset composition strategy and evaluation metrics are also extensible to related hygiene compliance tasks, including hand hygiene monitoring, contamination risk assessment, and workflow auditing in controlled environments.

A limitation of this study is that all experiments were conducted on Colab GPU rather than on embedded edge hardware. Nevertheless, the study was explicitly designed to be deployment-aware: we focused on nano-scale models (YOLOv8n, YOLOv10n, YOLOv12n) chosen for their suitability in resource-constrained IoT environments, and we reported efficiency metrics—Frames Per Second (FPS), parameter count, and GFLOPs—alongside accuracy measures to approximate edge device constraints. While these benchmarks provide practical guidance, they do not replace full end-to-end IoT deployment tests.

Future work will therefore emphasize live deployments of nano-scale detectors on edge devices (e.g., Raspberry Pi, NVIDIA Jetson) to evaluate system behavior—including alert latency, false positive rates, and IoT integration overhead. Additional efforts will explore temporal modeling for sequential behavior tracking, violation prediction, and the design of real-time compliance dashboards for proactive risk management.

Addressing remaining challenges—such as visual occlusion, PPE variability, and class imbalance—will be critical to improving detection robustness. These efforts support the broader vision of intelligent, lightweight compliance systems that scale reliably across diverse domains while minimizing false negatives in safety-critical workflows.

## 6. Conclusions

This study systematically evaluated three nano-scale object detectors: YOLOv8n, YOLOv10n, and YOLO12n, for real-time hygiene compliance monitoring in IoT-integrated environments. Using a curated dataset of over 31,000 annotated images spanning seven PPE-related classes, we assessed each model using both detection accuracy metrics (mAP@50, mAP@50–95, precision, recall) and deployment-relevant metrics (FPS, parameter count, and GFLOPs). Among the models tested, YOLOv10n offered the most balanced trade-off between accuracy and efficiency, achieving the highest mAP@50 (85.7%) and fastest inference speed (381.15 FPS), while maintaining a compact architecture suitable for edge deployment. YOLOv8n demonstrated the strongest localization accuracy at stricter IoU thresholds (mAP@50–95 = 55.7%) and the highest recall (80.7%), making it particularly robust for detecting subtle PPE violations, though at a modestly higher computational cost. YOLOv12n, despite being the smallest model (2.5M parameters, 5.8 GFLOPs), underperformed in both accuracy and inference speed, underscoring the limitations of extreme parameter reduction for safety-critical compliance monitoring.

These findings address the research objectives by offering insights into model selection, class-wise detection challenges, and deployment-aware trade-offs in hygiene-critical domains. More broadly, the study contributes an empirical comparison of nano-scale object detectors for deployment-aware evaluation, introduces a domain-specific dataset, and highlights practical trade-offs that can guide the design of scalable, real-time compliance monitoring systems in resource-constrained environments. Ultimately, the results demonstrate that carefully selected nano-scale architectures can provide reliable and efficient PPE monitoring, laying the groundwork for intelligent, IoT-enabled compliance solutions that enhance safety and operational efficiency in hygiene-regulated settings.

## Figures and Tables

**Table 1:** Class Distribution in the Annotated Dataset.

Class Name	Instances	Images
Glove	19,769	8335
Hairnet	13,325	6991
Incorrect_mask	10,863	8578
Mask	15,050	7846
No_glove	15,354	7086
No_hairnet	11,770	5970
No_mask	13,907	9109

**Table 2:** Comparison of YOLOv8n, YOLOv10n, and YOLOv12n across detection accuracy, inference speed, and model complexity metrics on the test set.

Metric	YOLOv8n	YOLOv10n	YOLOv12n
Precision (%)	84.3	83.9	80.5
Recall (%)	80.7	79.8	74.7
mAP@50 (%)	85.6	85.7	80.8
mAP@50–95 (%)	55.7	54.8	49.2
Size (parameters)	3.0 M	2.7 M	2.5 M
GFLOPs	8.1	8.2	5.8
FPS	336.30	381.15	277.89

**Table 3:** Per-class detection performance of YOLOv10n on the test set.

Class	Images	Instances	Precision (%)	Recall (%)	mAP@50	mAP@50–95
All	3028	9563	83.9	79.8	0.857	0.548
Glove	799	1956	89.3	79.3	0.878	0.628
Hairnet	653	1189	90.3	93.8	0.954	0.683
Incorrect_mask	845	1085	81.9	78.8	0.846	0.545
Mask	703	1412	76.8	72.0	0.788	0.473
No_glove	711	1537	82.8	70.4	0.796	0.458
No_hairnet	545	1071	84.1	85.5	0.895	0.599
No_mask	899	1313	81.9	78.8	0.844	0.448

## Data Availability

All datasets used in this study were sourced from publicly available Roboflow projects. We curated a domain-specific PPE compliance dataset by harmonizing annotations and remapping class labels. The finalized dataset is publicly available at https://doi.org/10.5281/zenodo.16329852.
